# Phage therapy for *Clostridioides difficile* infection

**DOI:** 10.3389/fimmu.2022.1057892

**Published:** 2022-10-28

**Authors:** Kosuke Fujimoto, Satoshi Uematsu

**Affiliations:** ^1^ Department of Immunology and Genomics, Osaka Metropolitan University Graduate School of Medicine, Osaka, Japan; ^2^ Division of Metagenome Medicine, Human Genome Center, The Institute of Medical Science, The University of Tokyo, Tokyo, Japan

**Keywords:** *Clostridioides difficile*, bacteriophage, endolysin, enzyme active domain, cell wall-binding domain, metagenome

## Abstract

*Clostridioides difficile* is endemic in the intestinal tract of healthy people. However, it is responsible for many healthcare-associated infections, such as nosocomial diarrhea following antibiotic treatment. Importantly, there have been cases of unsuccessful treatment and relapse related to the emergence of highly virulent strains of *C. difficile* and resistance to antimicrobial agents. Fecal microbiota transplantation (FMT) is considered an effective therapy for recurrent *C. difficile* infection. However, its safety is of concern because deaths caused by antibiotic-resistant bacterial infections after FMT were reported. Therefore, the development of effective *C. difficile*-specific treatments is urgently needed. In this review, we summarize the importance of phage therapy against *C. difficile*, and describe a novel next-generation phage therapy developed using metagenomic data.

## Introduction

The intestinal tract is constantly exposed to commensal microbiota and food antigens. Recently, great advances in intestinal microbial analyses have identified disease-associated alterations of the intestinal environment, such as dysbiosis, and research on the intestinal microbiome is progressing rapidly worldwide. Under dysbiosis, some symbiotic commensal bacteria acquire virulence traits, proliferate, and become directly involved in the development and progression of disease (1). These bacteria are referred to as ‘‘pathobionts,’’ which are distinct from opportunistic pathogens.

*Clostridioides difficile* (formally *Clostridium difficile*), a Gram-positive, spore-forming anaerobic bacterium, was first reported as a pathogen in 1978 (2), and is the representative pathobiont of nosocomial diarrhea following antibiotic treatment. *C. difficile* has three well-characterized toxins (toxin A, toxin B, and binary toxin) at two distinct loci. Whereas toxin A and toxin B are associated with primary virulence factors for *C. difficile* infection (CDI), binary toxin is involved in the increased severity of CDI. The production of the three toxins was shown to be strain-specific. Interestingly, *C. difficile* is frequently encountered in the gut microbiota of healthy individuals without any symptoms of disease (3). However, dysbiosis induced by antibiotic usage or malnutrition can initiate CDI with increasing *C. difficile* toxin concentrations, and *C. difficile* colonization and biofilm formation in the gut, resulting in severe disease status (4). *C. difficile* spores are highly resistant to environmental stresses such as high temperature, antibiotics, and disinfectants, which allows *C. difficile* to spread easily between residents of elderly care facilities (5).

In cases of mild CDI, withdrawing broad spectrum antibiotics such as ampicillin, clindamycin, and third generation cephalosporins is a standard therapy. Also, for cases of severe and recurrent CDI, use of the antibiotics vancomycin and fidaxomicin is an effective treatment (6). Recently, however, FMT has become an alternative therapy (7) and was reported to be successful with a cure rate of approximately 93% in patients with recurrent CDI. This is now accepted as a valid alternative for those failing antibiotic treatment (8). However, two deaths caused by antibiotic-resistant bacterial infections after FMT have been reported (9, 10), suggesting that a modification of FMT or alternative treatments are required to resolve safety concerns about FMT.

Although we are currently facing an unprecedented pandemic caused by severe acute respiratory syndrome-coronavirus-2, we must not forget that the threat of drug-resistant bacteria, including in *C. difficile*, remains a significant problem. If this trend continues, it is predicted that infections caused by drug-resistant bacteria will be the leading cause of death by 2050 (11, 12). Phage therapy is considered an important development against drug-resistant bacteria, and expectations for phage therapy are growing worldwide (13). This review summarizes recent findings in our understanding of CDI, with a particular focus on phage therapy for *C. difficile*.

## Phage therapy

Bacteriophages are viruses that infect bacteria and exhibit bactericidal effects against Gram-positive and Gram-negative bacteria. In 1896, Ernest Hankin discovered “an antiseptic substance” in the water of the Ganges River that killed certain bacteria. Later, in 1915, Frederick Twort discovered “a transparent material” that changed the properties of *Staphylococcus aureus*, and in 1917 (14), Félix d’Hérelle named an “invisible microbe” that dissolved dysentery bacteria as a “bacteriophage”. Furthermore, d’Hérelle advocated “phage therapy” to treat bacterial infections, and practiced this in humans and animals, including those with dysentery and Vibrio cholerae. d’Hérelle met Giorgi Eliava at the Pasteur Institute, which led to the establishment of several institutes in Tbilisi, Georgia, between 1923 and 1936 with the support of the Soviet Union. One of these, the Eliava Institute, became an important focus for phage research and therapy, and remains the core research institute for phage research today. However, after the discovery of antibiotics, especially penicillin, in 1928 (15) and its subsequent clinical application, phage therapy was abandoned in Western countries. However, during the Cold War, phage therapy was developed as an alternative treatment for infectious diseases in the Soviet Union because of the lack of newly developed antibiotic drugs. Even today, phage cocktails are prescribed and used as an effective treatment for infectious diseases in Russia, Georgia, Poland, and other countries (16). The overuse of antibiotics has led to the emergence of various multidrug-resistant bacteria, which has become a serious medical problem worldwide. Therefore, phage therapy against multidrug-resistant bacteria is beginning to attract attention as a next-generation treatment method.

## Phages against *Clostridioides difficile*

*C. difficile*-specific phages have been investigated for the regulation of *C. difficile* pathogenesis and development of new therapies for CDI. Many *C. difficile*-specific phages have been identified to date [phiC2 (17, 18), phiC5 (17), phiC6 (17), phiC8 (17), phiCD119 (19), phiCD24-1 (20), phiCD27 (21), phiCD6356 (22), phiCD6365 (22), phiCD38-2 (23), phiMMP01 (24, 25), phiMMP02 (24, 25), phiMMP03 (24, 25), phiMMP04 (24, 25), phiCD24-2 (25), phiCD146 (25), phiCD111 (25), phiCD526 (25), phiCD52 (25), phiCD481-1 (25), phiCD481-2 (25), phiCD505 (25), phiCD506 (25), phiCD508 (25), phiCDHM1 (26), phiCDHM2 (26), phiCDHM3 (26), phiCDHM4 (26), phiCDHM5 (26), phiCDHM6 (26), phiCDHS1 (26), CDKM9 (27), CDKM15 (27), phiSemix9P1 (28), phiCD5763 (29), phiCD5774 (29), phiCD2955 (29), phiCD211/phiCDIF1296T (30), phiHN10 (31), phiHN16-1 (31), phiHN16-2 (31), phiHN50 (31), JD032 (32), and phiCDKH01 (33)] (**Supplementary Table S1**). The host for all of these phages is *C. difficile*; however, some were shown to infect multiple *C. difficile* strains. In addition, the *C. difficile*-specific phages described above are lysogenic, not lytic, and they are classified in the *Myoviridae* or *Siphoviridae* subfamilies of Caudovirales. Because lytic phages against *C. difficile* should exist, the fact that *C. difficile* is anaerobic and forms spores makes it difficult to isolate lytic phages experimentally.

The application of phage targeting *C. difficile* should be considered. Indeed, the efficacy of phage monotherapy against *C. difficile* has been studied for the past decade (26, 34–37). Unfortunately, none of these studies have led to the development of effective treatments for CDI. Simply identifying and isolating *C. difficile*-specific phages by conventional methods using ultraviolet or mitomycin C has not led to successful phage therapy against *C. difficile* (17–23, 31). Therefore, it was considered necessary to develop a new lysis method that takes advantage of the unique properties of phages.

## Phage-derived endolysins against *Clostridioides difficile*

Endolysin, an enzyme encoded by phages, is used by mature phage virions to hydrolyze the bacterial cell wall from the inside. The worldwide increase in antibiotic-resistant bacteria has stimulated research on endolysins as an alternative therapeutic agent. Endolysins can degrade peptidoglycans from the outside, and also have the potential to lyse bacterial biofilms (38). Therefore, the medical applications of endolysin may provide new treatment options for Gram-positive bacterial infections.

**Figure 1:**
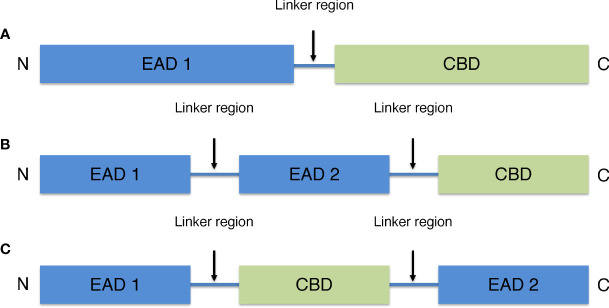
General schematic models of endolysins derived from phages that infects Gram-positive host. **(A)** Model with one N-terminal EAD and one C-terminal CBD. **(B)** Multi-domain model with two EAD and one C-terminal CBD. **(C)** Multi-domain model with one CBD located between two EAD.

Most endolysins derived from phages that infect Gram-positive hosts are modular (39). Their molecular weight is typically 15–40 kDa. They are characterized by one or two (multi-domain) N-terminal enzyme active domains (EAD) often linked to a C-terminal cell wall-binding domain (CBD) by a short flexible linker region. The N-terminal EAD of modular endolysins cleaves various specific peptidoglycan bonds in the host bacterial murein layer, whereas the C-terminal CBD recognizes and binds to different structures in the cell wall to properly anchor the catalytic effect of the EAD (**Figure 1**) (40). The endolysins of phages that infects Gram-positive host are structurally similar to those of fungal cellulases (41).

There are four types of EAD including amidases, acetylmuramidases, endopeptidases, and glucosaminidases. Amidases break the amide bond between peptide and sugar chains. Acetylmuramidases break the N-acetylmuramoyl β1,4 N-acetylgulcosamine bond of sugar strands. Endopeptidases break the peptide crosslinking in the stem structure of peptidoglycans and glucosaminidases break the N-acetylmuramoyl β1,4 N-acetylmuramine glycosidic bond. Among endolysins, amidases and muramidases are the most common endolysins that target the highly conserved binding of peptidoglycans.

CBD binds to murein ligands and secondary cell wall polymers, which are cell wall components that include teichoic acid and natural polysaccharides of bacteria (42). The main function of CBD is to provide endolysins with host specificity for non-covalent binding to cell membrane ligands. For example, LysM, the most widely reported endolysin, binds to N-acetylglucosamine in the peptidoglycan glycan backbone (43, 44). The CBD motif in the endolysin of many phages that infects Gram-positive host might allow this domain to bind to ligands in the peptidoglycan layer, preventing endolysin diffusion and destroying the nearby host after cell lysis (39). Because EAD do not have specificity for bacterial cell walls, CBD are considered important in defining their specificity for target cells. Although many endolysins retain or increase bacteriolytic activity in the absence of CBD, host specificity is very important when endolysins are used as a treatment for multidrug-resistant bacteria, for example.

As well as other pathogens, the application of phage-derived endolysins targeting *C. difficile* has been explored [CD27L (21), phyCD (45), CDG (46), CD11 (46), LCD (47), and CWH (48)]. Among these, CD27L derived from phiCD27 was first identified as a *C. difficile*-specific endolysin. CD27L lyses 30 C*. difficile* strains, including two strains of the hypervirulent ribotype 027 (21). Importantly, a range of commensal species that inhabited the gastrointestinal tract was insensitive to the endolysin (21, 49), indicating that CD27L is not expected to induce gut dysbiosis. This endolysin might provide a platform for the generation of novel therapeutic agents to overcome *C. difficile*.

## Metagenome data-based next-generation phage therapy for CDI

The future practice of phage therapy for CDI is promising. However, it is difficult to identify phages that have *C. difficile* as a host. Given the versatility of phage therapy for CDI, there is a need for a high-throughput system that can identify phages that infect *C. difficile*.

**Figure 2:**
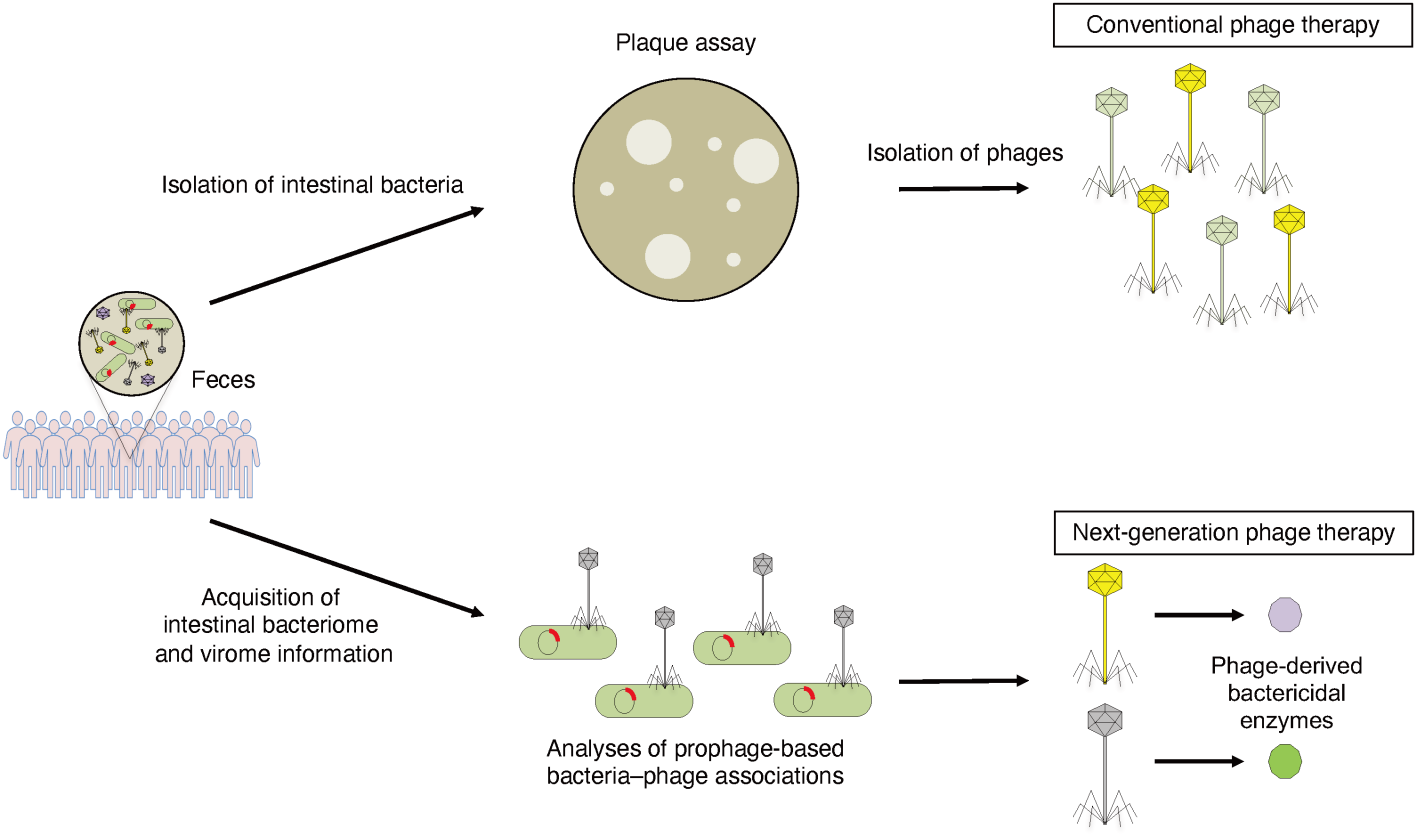
Conventional phage therapy and next-generation phage therapy based on metagenomic information. To isolate phages that can specifically control pathobionts, host bacteria is isolated from human fecal samples and plaque assay is performed. Isolated phages are used for phage therapy (conventional phage therapy). To detect phage-derived antibacterial enzymes that can specifically regulate pathobionts, intestinal bacterial and viral metagenomic information is acquired from human fecal samples. Phage-derived bactericidal enzymes can kill host bacteria specifically (next-generation phage therapy).

Recently, to develop phage therapies to control pathobionts, which are directly involved in the pathogenesis of disease, we established an effective method for obtaining genomic information on host bacteria–phage associations (50, 51). Metagenomic data obtained from the fecal samples of healthy subjects and clinical isolates of *C. difficile* strains were used to develop a phage therapy specific for *C. difficile*. The obtained sequencing data were used to search for novel phage-derived endolysins specific for *C. difficile*. Using the phage genome analysis pipeline, we identified several novel endolysin sequences from the prophage sequence of *C. difficile*. These endolysins were synthesized and shown to exhibit bacteriolytic activity *in vitro* and to be effective in a mouse model of CDI (50). This is a practical example of a next-generation phage therapy based on metagenomic information. To isolate phages used in phage therapy, it has been necessary to isolate and culture target bacteria. Phages could not be isolated for non-culturable bacteria in the intestine. Thus, this strategy is not limited to *C. difficile* and might be applied to various targeted bacteria in the future (**Figure 2**).

## Conclusion

Scientific research on bacteriophages has become a hot topic because of the impending problem of multidrug-resistant bacteria. Because the development of antibiotics is not expected to progress as far as expected, it is hoped that phage therapy will become commercially available.

With improvements in genome analysis technology using next-generation sequencing, the analysis of intestinal microbiota has progressed dramatically, and its relationship to disease has gradually become clearer. It is now possible to analyze intestinal phages, which has been difficult in the past, and this is expected to become an extremely powerful analytical tool for the future practice of phage therapy, as well as leading to various industrial applications of phages. Recently, Federici et al. have demonstrated the feasibility of combination phage therapy for pathobionts associated with inflammatory bowel disease (52). It would be an interesting direction to further phage therapies for other intestinal bacteria-mediated diseases including CDI. In the near future, phage science will be developed further by integration with a wide range of fields including medicine, microbiology, bioinformatics, and synthetic biology.

## Author contributions

All authors listed have made a substantial, direct, and intellectual contribution to the work and approved it for publication.

## Funding

This study was supported by the Japan Agency for Medical Research and Development (AMED) (to KF; 22ae0121048h0001 and to SU; 22fk0108619h0001), and Grant-in-Aid for Early-Career Scientists (to KF; 22K16329) from the JSPS.

## Acknowledgments

We thank K. Ogawa, M. Maeda, and K. Suetsugu for administrative assistance. We thank Edanz Group (https://jp.edanz.com/ac) for editing a draft of this manuscript.

## Conflict of interest

The authors declare that the research was conducted in the absence of any commercial or financial relationships that could be construed as a potential conflict of interest.

## Publisher’s note

All claims expressed in this article are solely those of the authors and do not necessarily represent those of their affiliated organizations, or those of the publisher, the editors and the reviewers. Any product that may be evaluated in this article, or claim that may be made by its manufacturer, is not guaranteed or endorsed by the publisher.
