# Supplementary material for: Phage therapy for Clostridioides difficile infection
Source: Front Immunol. 2022 Oct 28;13:1057892. doi: 10.3389/fimmu.2022.1057892 (PMC9650352; doi:10.3389/fimmu.2022.1057892)
Supplement: Supplementary file 1 [file Table_1.pdf]

Supplementary Table 1. Characterizations of *C. difficile* phages.

Phage	Family	Grouth cycle	Induced *C. difficile* strains	*C. difficile* sensitive strains (Ribotype)	Reference
phiC2	*Myoviridae*	Lysogenic	CD242	-	(17,18)
phiC5	*Myoviridae*	Lysogenic	CD578	-	(17)
phiC6	*Siphoviridae*	Lysogenic	CD371	-	(17)
phiC8	*Myoviridae*	Lysogenic	CD371	-	(17)
phiCD119	*Myoviridae*	Lysogenic	CD602	-	(19)
phiCD24-1	*Siphoviridae*	Lysogenic	CD24	-	(20)
phiCD27	*Myoviridae*	Lysogenic	-	NCTC11204/NCTC11205/NCTC11207/NCTC11209	(21)
phiCD6356	*Siphoviridae*	Lysogenic	-	-	(22)
phiCD6365	*Siphoviridae*	Lysogenic	-	-	(22)
phiCD38-2	*Siphoviridae*	Lysogenic	CD38	CD73/CD77/CD105/CD111 (027)/CD192 (027)/CD274 (027)/CD316/CD337/CD383 (027)/CD384/CD398/CD419 (027)/CD420 (027)/CD425 (001)/CD426 (106)/CD427 (001)/CD430 (002)/CD540/CD475/CD481 (014)/CD490 (002)/CD505 (014)/CD515	(23)
phiMMP01	*Myoviridae*	Lysogenic	-	CD19/CD93 (012)/CD117 (014)/CD125/CD211 (001)/CD273 (037)/CD326 (078)/CD384/CD425 (001)/CD426 (106)/CD427 (001)/CD429 (014)/CD544/CD511	(24, 25)
phiMMP02	*Myoviridae*	Lysogenic	CD343	CD117 (014)/CD426 (106)/CD429 (014)/CD481 (014)/CD511/CD515	(24, 25)
phiMMP03	*Myoviridae*	Lysogenic	CD368	CD117 (014)/CD505 (014)	(24, 25)
phiMMP04	*Myoviridae*	Lysogenic	CD380	CD19/CD24 (078)/CD73/CD93 (012/CD105/CD316/CD337/CD427 (001)/CD428 (015)/CD430 (002)/CD490/CD515	(24, 25)
phiCD24-2	*Myoviridae*	Lysogenic	CD24	CD19/CD73/CD93 (012)/CD117 (014)/CD125/CD211 (001)/CD273 (037)/CD326 (078)/CD384/CD425 (001)/CD427 (001)/CD429 (014)/CD544/CD493 (078)/CD511	(25)
phiCD146	*Siphoviridae*	Lysogenic	CD146	CD19/CD77/CD105/CD111 (027)/CD192/CD211 (001)/CD274 (027)/CD316/CD337/CD383 (027)/CD419 (027)/CD420 (027)/CD427 (001)/CD430 (002)/CD540/CD475/CD490/CD515	(25)
phiCD111	*Siphoviridae*	Lysogenic	CD111	CD77/CD125/CD192/CD211/CD273/CD274/CD420/CD425/CD426/CD427/CD540/CD475/CD490 (002)/CD515	(25)
phiCD526	*Myoviridae*	Lysogenic	CD526	CD117 (014)/CD316/CD490 (002)/CD505 (014)	(25)
phiCD52	*Myoviridae*	Lysogenic	CD52	CD24 (078)/CD73/CD384/CD398	(25)
phiCD481-1	*Myoviridae*	Lysogenic	CD481	CD117 (014)/CD118/CD337/CD419 (027)/CD515	(25)
phiCD481-2	*Myoviridae*	Lysogenic	CD481	CD73/CD118/CD337/CD515	(25)
phiCD505	*Myoviridae*	Lysogenic	CD505	CD19/CD117 (014)/CD326 (078)/CD481 (014)/CD511	(25)
phiCD506	*Myoviridae*	Lysogenic	CD506	CD493 (078)	(25)
phiCD508	*Myoviridae*	Lysogenic	CD508	CD117 (014)/CD426 (106)/CD430 (002)/CD505 (014)	(25)
phiCDHM1	*Myoviridae*	Lysogenic	-	CD66 (014/020)/CD81 (015)/CD89 (015)/CD105LC2 (014/020)/CD106 (106)/AUS1036 (002)/LEEDS003 (003)/AQV (003)/ATJ (014/020)/ATK (014/020)/TL176 (014/020)/ANS (014/020)/LEEDS018 (018)/AKL (023)/AJX (023)/LV22 (106)	(26)
phiCDHM2	*Myoviridae*	Lysogenic	-	CD66 (014/020)/CD81 (015)/CD89 (015)/CD105LC2 (014/020)/CD106 (106)/AUS1036 (002)/AIL (002)/AIJ (002)/ATH (002)/LEEDS003 (003)/AQV (003)/2007831 (003)/AOO (005)/AKR (013)/ARS (013)/ATJ (014/020)/ATK (014/020)/TL176 (014/020)/ANS (014/020)/LEEDS018 (018)/LV22 (106)/M322630 (127)	(26)
phiCDHM3	*Myoviridae*	Lysogenic	-	CD66 (014/020)/CD81 (015)/CD89 (015)/CD105LC2 (014/020)/CD106 (106)/AUS1033 (002)/AUS1036 (002)/AIL (002)/AIJ (002)/TL178 (002)/ATH (002)/2007831 (003)/AKR (013)/ARS (013)/ATJ (014/020)/ATK (014/020)/TL176 (014/020)/AUS1022 (014/020)/ANS (014/020)/ATO (015)/LEEDS018 (018)/AKL (023)/AJX (023)/LEEDS023 (023)/AJV (027)/AKM (078)/AKP (078)/ALL (078)/APX (087)/LV22 (106)/M322630 (127)	(26)
phiCDHM4	*Myoviridae*	Lysogenic	-	2007831 (003)/10 (012)/LEEDS018 (018)/APT (087)	(26)
phiCDHM5	*Myoviridae*	Lysogenic	-	CD66 (014/020)/CD81 (015)/CD89 (015)/CD105LC2 (014/020)/AUS1036 (002)/AIL (002)/AOO (005)/AKR (013)/ARS (013)/ATJ (014/020)/ATK (014/020)/TL176 (014/020)/ANS (014/020)/ATO (015)/LEEDS018 (018)/AKL (023)/AJX (023)/ALN (026)/LV22 (106)/M322630 (127)	(26)
phiCDHM6	*Myoviridae*	Lysogenic	-	CD66 (014/020)/CD81 (015)/CD89 (015)/CD105LC2 (014/020)/AIL (002)/AIJ (002)/ATH (002)/2007831 (003)/AOO (005)/AKR (013)/ARS (013)/ATJ (014/020)/ATK (014/020)/TL176 (014/020)/AUS1022 (014/020)/ANS (014/020)/ATU (015)/ATR (015)/LEEDS018 (018)/AKL (023)/AJX (023)/M322630 (127)	(26)
phiCDHS1	*Siphoviridae*	Lysogenic	-	CD001 (001)/CD196 (027)/AUS1025 (001)/AUS1021 (001)/AUSCD84 (001)/AIP (001)/AQV (003)/2007831 (003)/AOY (005)/AKR (013)/ARS (013)/ALV (015)/LEEDS018 (018)/UK023 (023)/LEEDS023 (023)/AUS1032 (027)/AUS1024 (027)/R20291 (027)/B19 (027)/AJS (027)/AJV (027)/AMJ (027)/AMZ (027)/ANB (027)/US027 (027)/2006237 (027)/ANO (081)/ANQ (081)/APT (087)/ASQ (107)	(26)
CDKM9	*Myoviridae*	Lysogenic	-	CD105HS23 (001)/CD105HS25 (001)/CDNCTC11204 (001)/CD105KSE3 (001)/F1 (001)/CD105HS10 (005)/CD105LI07 (010)/CD105HS33 (010)/CD105HS14 (010)/CD105HS4 (014)/CD105HS42 (014)/CD105HS39 (015)/CD105LC27 (014/020)/CD105LC278 (014/020)/CD105LC1 (027)/CD105HS8 (027)/CD105HS35 (031)/CD105KSE11 (035)/CD105KSE5 (035)/CD105HE1 (076)/CD106 (106)/CD105HS46 (106)/R39V106 (106)/CD105HS6 (220)/CD105HS12 (220)	(27)
CDKM15	*Myoviridae*	Lysogenic	-	CDNCTC11204 (001)/CD105KSE3 (001)/F1 (001)/CD105HS20 (005)/CD105LI07 (010)/CD105HS4 (014)/CD105HS42 (014)/CD105LC27 (014/020)/CD105LC278 (014/020)/CD105LC1 (027)/CD105HS8 (027)/CD105HE1 (076)/CD106 (106)/R40V0106 (106)/R6106 (106)/CD105HS46 (106)/R39V106 (106)/CD105HS22 (220)/CD105HS6 (220)/CD105HS12 (220)	(27)
phiSemix9P1	*Myoviridae*	Lysogenic	Semix9	-	(28)
phiCD5763	*Siphoviridae*	Lysogenic	LIBA-5763	-	(29)
phiCD5774	*Siphoviridae*	Lysogenic	LIBA-5774	-	(29)
phiCD2955	*Siphoviridae*	Lysogenic	LIBA-2955	-	(29)
phiCD211/phiCDIF1296T	*Siphoviridae*	Lysogenic	DSM1296^{T}/ATCC9689/CD211	-	(30)
phiHN10	*Myoviridae*	Lysogenic	HN10	CD630 (012)/HR118 (017)/HN2 (017)/HN6 (017)/HN9 (017)/HN21 (017)	(31)
phiHN16-1	*Myoviridae*	Lysogenic	HN16	HN21 (017)	(31)
phiHN16-2	*Myoviridae*	Lysogenic	HN16	HN21 (017)	(31)
phiHN50	*Myoviridae*	Lysogenic	HN50	HN21 (017)	(31)
JD032	*Myoviridae*	Lysogenic	TW69	-	(32)
phiCDKH01	*Siphoviridae*	Lysogenic	CD34-Sr	-	(33)
